# Walks with Small Steps in the 4D-Orthant

**DOI:** 10.1007/s00026-020-00520-5

**Published:** 2021-03-10

**Authors:** Manfred Buchacher, Sophie Hofmanninger, Manuel Kauers

**Affiliations:** Institute for Algebra, J. Kepler University Linz, Linz, Austria

## Abstract

We provide some first experimental data about generating functions of restricted lattice walks with small steps in ${\mathbb {N}}^4$.

## Introduction

A lattice walk is a sequence of points $P_0,P_1,\dots ,P_n$ in $\mathbb {Z}^d$. The points $P_0$ and $P_n$ are its starting and end points, respectively, the consecutive differences $P_{i+1}-P_i$ its steps, and *n* is its length. Given a set $S\subseteq {\mathbb {Z}}^d$, called the step set, and a set $D\subseteq \mathbb {Z}^d$, called the domain, and elements *P* and *Q* of *D*, we are interested in the number *a*(*P*, *Q*; *n*) of walks of length *n* that start at *P*, have all their steps in *S*, have all their points in *D*, and end at *Q*. Is there a simple formula in terms of the coordinates of the end point and the length of the walks, and if not, can we at least say something about the asymptotic behaviour of these numbers as *n* goes to infinity? A first step towards answering these questions can be done by considering the generating function:

$$\begin{aligned} f(x,t) = \sum _{n\ge 0}\biggl (\sum _{Q\in D} a(P,Q;n) x^Q\biggr ) t^n\in \mathbb {Q}(x)[[t]] \end{aligned}$$

that is associated with these numbers and determining whether it has one of the following two properties:

### Definition 1

Let *C* be a field.

1. A series $f\in C((t))$ is called algebraic if there are polynomials $p_0,\dots ,p_{r-1}$ and a non-zero polynomial $p_r$ in *C*[*t*], such that $p_0(t) + p_1(t)f(t) + \cdots + p_r(t)f(t)^r = 0$.
2. A series $f\in C((t))$ is called D-finite if there are polynomials $p_0,\dots ,p_{r-1}$ and a non-zero polynomial $p_r$ in *C*[*t*], such that $p_0(t)f(t) + p_1(t) f'(t) + \dots + p_r(t) f^{(r)} = 0$.

It is well known that every algebraic series is D-finite, but not vice versa. Knowing that a formal power series is algebraic, or D-finite, not only allows a finite representation of and basic operations to be performed effectively on it, but also makes available a variety of algorithms dealing with tasks ranging from the fast computation of their coefficients and determining their asymptotic behaviour to deciding whether there is a simple formula for them.

For $D={\mathbb {Z}}^2$ or $D={\mathbb {Z}}\times {\mathbb {N}}$, the generating function is always algebraic, regardless of the choice of *S*, see [6, Proposition 18], but for $D={\mathbb {N}}^2$, it was observed by Bousquet-Mélou and Mishna [11] that the nature of the generating function does depend on the step set. Even if we restrict the step sets to subsets of $\{-1,0,1\}^2$, sets of so-called small steps, we find that for some step sets the generating function is algebraic; for others, it is not algebraic but still D-finite, and for yet others, it is not even D-finite. This observation sparked an intensive research activity to which many authors have contributed, see [2, 4, 7, 9, 12, 13, 15, 20] for some of the milestones and for further references. As a result of this work, the classical setting of walks in the quarter plane is relatively well understood, and the focus of interest is now shifting to the study of variations and generalizations. One such generalization concerns the situation in higher dimensions. A first step was taken by Bostan and Kauers in [8], who used automated guessing to identify potentially D-finite step sets of size up to 5 in three dimensions. This work was extended by Bostan, Bousquet-Mélou, Kauers, and Melczer [5] to step sets of size up to 6. They introduced the notions of dimension of a lattice walk model and Hadamard decomposition of a step set, which allow to reduce some of the problem to walks in lattices of lower dimension, and they used these new concepts as well as the classical orbit sum method for proving D-finiteness in certain cases. Bacher, Kauers, and Yatchak [1] have extended this work to step sets of arbitrary size, Du, Hou, and Wang provided non-D-finiteness results for many cases [14], and most recently, Bogosel, Perrollaz, Raschel, and Trotignon [3] have systematically explored the asymptotic behaviour of counting sequences for walks in the octant and observed a striking relation between the nature of the generating function and the angles of certain triangles on the sphere. Despite all this progress, there are still many open questions related to walks in the octant. In particular, there is a list of 170 models whose nature remains unclear. For example, this list includes the 3D version of the classical 2D Kreweras model [10, 11, 19], the step set $\{(-1,0,0),(0,-1,0),(0,0,-1),(1,1,1)\}$. Although the 2D version has an algebraic generating function, the current asymptotic estimates suggest (without proof) that the 3D version is not D-finite.

In this short note, we have nothing new to say about the 3D cases. Instead, our aim is to open the discussion for 4D. When the dimension of the lattice increases, the classification problem becomes more difficult in two ways. First, and most importantly, the total number of models explodes. For dimension *D*, there are $2^{3^D-1}$ step sets, which evaluates to more than $10^{24}$ when $D=4$. There is no way to go through all of them in a reasonable time, even if we spend only a tiny amount of computation time per model. The second problem is that it will not be enough to spend only a tiny amount of computation time per model, because with increasing dimension, it also becomes more costly to analyze a particular model. For example, computing the first *N* terms of a counting sequence using the standard recurrence requires $\mathrm {O}(N^{D+1})$ time and $\mathrm {O}(N^D)$ memory. For $D=4$, this means that on a computer with 1 Tb of main memory, we were only able to compute $N=700$ terms of a counting sequence.

## Search Procedure

To identify potentially interesting models, we have applied a similar search procedure as Bacher, Kauers, and Yatchak [1] did in their search for interesting models in 3D. The procedure can be summarized as follows:

### Only Step Sets $S\subseteq \{-1,0,1\}^4\setminus \{(0,0,0,0)\}$ with $|S|\le 7$ or $|S|\ge 73$ Were Considered

This restriction has no combinatorial motivation, but was only made to reduce the computational cost to a manageable amount, similar as it was done in [5, 8] for 3D. Note that the number

$$\begin{aligned} \sum _{k=0}^7\left( {\begin{array}{c}3^4-1\\ k\end{array}}\right) +\sum _{k=73}^{80}\left( {\begin{array}{c}3^4-1\\ k\end{array}}\right) =7005847194\approx 7\cdot 10^9 \end{aligned}$$

of remaining models is still quite big (though of course much smaller than $2^{3^4-1}\approx 1.2\cdot 10^{24}$).

### Step Sets Containing Unused Steps were Discarded

Recall from [5] that an element *s* of *S* is called unused if it cannot appear in any walk of the model. For example, the step set $S=\{(1,0,-1,0),(0,1,0,-1),(1,1,0,0)\}$ leads to the same generating function as the step set $\{(1,1,0,0)\}$, because any use of $(1,0,-1,0)$ or $(0,1,0,-1)$ would lead the walk out of ${\mathbb {N}}^4$, which is not allowed.

To check whether a given step set $S\subseteq \{-1,0,1\}^D$ contains unused steps, we successively determine the ‘not unused’ steps, i.e., the steps which can occur in a walk. We start with the elements of the step set that belong to $\{0,1\}^D$. Any of these steps can be the first step of a walk in the model, and every walk in the model must start with one of these steps. The walks built only from these steps can proceed arbitrarily far into a certain direction $d\in \{1,\dots ,D\}$ for which there is a step $(s_1,\dots ,s_D)\in S\cap \{0,1\}^D$ with $s_d=1$. Set $u_d=\mathrm {true}$ for these *d* and $u_d=\mathrm {false}$ for all other *d*. We can next recognize all steps of *S* as ‘not unused’ which only have negative entries in coordinate *d* for which $u_d$ is true. For example, if $u_1$ is $\mathrm {true}$, then $(-1,1,0,0)$ is not an unused step. For $i=1,\dots ,D$, we update $u_d$ to true if any of these additional steps has a positive *d*th coordinate. With the updated values of $u_1,\dots ,u_D$, we can check whether further elements of *S* can be recognized as ‘not unused’. If so, we update $u_1,\dots ,u_D$ again. We repeat the process until the step of recognized ‘not unused’ steps is saturated. The step set *S* contains unused steps if and only if the set of recognized ‘not unused’ steps is a proper subset of *S*.

### Only One Step Set from Each Symmetry Class was Considered

Permuting the coordinates of the steps in a step set amounts to permuting the variables of the corresponding generating function. For example, if $f(x_1,x_2,x_3,x_4,t)$ is the generating function of the model with step set $\{(1,0,1,1),(-1,1,0,0),$ $(0,0,0,1)\}$, then $f(x_2,x_4,x_1,x_3,t)$ is the generating function for the model with step set $\{(0,1,1,1),(1,0,-1,0),$ $(0,1,0,0)\}$. Since permutation of variables preserves algebraicity and D-finiteness, it suffices to consider one model per equivalence class. This filter reduces the number of cases to be considered by roughly a factor of $D!=24$.

For deciding whether two step sets $$\{s_1,\dots ,s_m\},\{s_1',\dots ,$$$$s_m'\}\subseteq \{-1,0,1\}^D$$ are equivalent, we need to decide whether there is a permutation $$\pi \in S_D$$, such that $$\{s_1,\dots ,s_m\}=\{\pi \cdot s_1',\dots ,\pi \cdot s_m'\}$$, where $$\pi \cdot s_i'$$ denotes the tuple obtained from $$s_i'$$ by permuting its coordinates according to $$\pi $$. Since *D* is small, we can simply test this by trying out all $$\pi \in S_D$$. However, what we really need is not a method for checking whether two given step sets are equivalent: rather, when we go through all the step sets, we are rather in the situation that we have a single step set at hand and have to decide whether we should consider it or not. We do this by defining a total order on the step sets and rejecting a step set $$\{s_1,\dots ,s_m\}$$ whenever there is a $$\pi \in S_D$$, such that $$\{\pi \cdot s_1,\dots ,\pi \cdot s_m\}$$ is smaller in the chosen order.

### Step Sets Admitting a Hadamard Decomposition Were Discarded

Recall from [5] that a step set $S\subseteq \{-1,0,1\}^D$ is said to admit a *d*-Hadamard decomposition for some $d\in \{1,\dots ,D-1\}$ if it can be written as $S=(V\times \{0\}^{D-d})\cup (U\times W)$ with $V,U\subseteq {\mathbb {Z}}^{d}$ and $W\subseteq {\mathbb {Z}}^{D-d}\setminus \{0\}$. If this is the case, the generating function for the lattice walk model for *S* can be expressed in terms of the Hadamard product of the generating functions associated with the lower dimensional models corresponding to *W* and a model with step set $(U\times \{0\})\cup (V\times \{1\})$. As the D-finiteness of models admitting a Hadamard decomposition can be easily explained (see the explanation in Sect. 5 of [5] for details), we discard them from consideration.

It is easy to decide whether a given step set *S* is *d*-Hadamard. Write $\pi _1:{\mathbb {R}}^D\rightarrow {\mathbb {R}}^d$ for the projection on the first *d* coordinates and $\pi _2:{\mathbb {R}}^D\rightarrow {\mathbb {R}}^{D-d}$ for the projection to the last $D-d$ coordinates. Set $V = \{s\in S:\pi _2(s)=0\}$, $U = \pi _1(S\setminus V)$, $W=\pi _2(S\setminus V)$ and check whether $S=(V\times \{0\}^{D-d})\cup (U\times W)$. We need to observe however that the definition of Hadamard decomposition as quoted implicitly assumes a particular ordering of the coordinates. For example, while

$$\begin{aligned}&\{(1,0,0),(-1,0,0)\}\cup (\{-1,1\}\times \{(1,-1)\})\\&\quad =\{(1,0,0),(-1,0,0),(-1,1,-1),(1,1,-1)\} \end{aligned}$$

is 1-Hadamard, the equivalent step set $$\{(0,1,0),(0,-1,0),$$$$(1,-1,-1),(1,1,-1)\}$$ strictly speaking is not. Our program filters out all step sets which by a suitable permutation of coordinates can be mapped to a Hadamard model. It does so by simply carrying out the test sketched above for all elements in the orbit of the step set under consideration.

### Step Sets with Dimension Less than Four Were Discarded

Recall from [5] that the dimension of a model is defined as the number of coordinates for which the non-negativity restriction is not redundant. For example, for the step set $\{(1,1,1),(1,-1,0),(1,0,-1)\}$, the number of walks in ${\mathbb {N}}^3$ is the same as the number of walks in ${\mathbb {Z}}\times {\mathbb {N}}^2$, because there is no way to get a negative first coordinate with the available steps. As the restriction on the other two coordinates is essential, the dimension is 2 in this case. Since lattice walk models in ${\mathbb {N}}^4$ whose dimension is less than 4 are equivalent to models in ${\mathbb {N}}^3$ (possibly with multiple steps), it is fair to discard them.

Whether a given step set $S=\{s_1,\dots ,s_m\}\subseteq \{-1,0,1\}^D$ has dimension less than *D* can be found out by linear programming. Writing $s_{i,j}$ for the *i*th coordinate of the *j*th step, the requirement is that there is some $i\in \{1,\dots ,D\}$, such that for all non-negative $x_1,\dots ,x_m\in {\mathbb {R}}$ with $\sum _{j=1}^m s_{k,j}x_j\ge 0$ for all $k\ne i$, we also have $\sum _{j=1}^m s_{i,j}x_j\ge 0$. Linear programming allows us to find the minimum value assumed by $\sum _{j=1}^m s_{i,j}x_j$ when $x_1,\dots ,x_m$ ranges over all non-negative real numbers with $\sum _{j=1}^m s_{k,j}x_j\ge 0$ for all $k\ne i$. If the minimum is 0, then the *i*th coordinate is redundant, and the model has dimension less than *D* and can be discarded. If none of the coordinates is recognized as redundant in this way, then the model has dimension *D*.

### Step Sets Whose Associated Group Has More than 800 Elements Were Discarded

Recall from [5, 11] that to every model of maximal dimension, we can associate a certain group. Given a step set $S\subseteq \{-1,0,1\}^D\setminus \{(0,\dots ,0)\}$, the group is constructed as follows. First, define the step set polynomial:

$$\begin{aligned} P_S:=\sum _{(s_1,\dots ,s_D)\in S} x_1^{s_1}\cdots x_D^{s_D} \end{aligned}$$

(also called the inventory by some authors). Then, for $i=1,\dots ,D$, let $\Phi _i$ be the rational map that sends $x_j$ to itself for $j\ne i$ and $x_i$ to $x_i^{-1}\frac{[x_i^{-1}]P_S}{[x_i]P_S}$, where $[x_i^{\pm 1}]P_S$ refers to the coefficient of $x_i^{\pm 1}$ in $P_S$ when $P_S$ is viewed as a Laurent polynomial in $x_i$ whose coefficients are Laurent polynomials in the remaining variables. The group associated to *S* is the group generated by $\Phi _1,\dots ,\Phi _D$ under composition.

For example, for $S=\{(-1,-1),(1,-1),(1,0),(-1,1), (1,1)\}$, we have $P_S=x_1^{-1}x_2^{-1} + x_1x_2^{-1} + x_1 + x_1^{-1}x_2 + x_1x_2$ and get:

$$\begin{aligned}&\Phi _1 = \begin{pmatrix} x_1\\ x_2 \end{pmatrix}\mapsto \begin{pmatrix} x_1^{-1}\frac{x_2+x_2^{-1}}{x_2+1+x_2^{-1}} \\ x_2 \end{pmatrix}\qquad \text {and}\\&\Phi _2 = \begin{pmatrix} x_1\\ x_2 \end{pmatrix}\mapsto \begin{pmatrix} x_1 \\ x_2^{-1} \end{pmatrix}, \end{aligned}$$

and the group $\langle \Phi _1,\Phi _2\rangle$ turns out to have only four elements: $\mathrm {id},\Phi _1,\Phi _2$ and $\Phi _1\circ \Phi _2$.

A main result about the case $D=2$ is that for full-dimensional models, this group is finite if and only if the generating function is D-finite [5, 7, 11]. While the experimental results for $D=3$ suggest that there may be non-D-finite cases with finite group, we are not aware of any (conjectured) D-finite case with an infinite group. For this reason, and also because a finite group gives the chance to apply the so-called orbit sum method for proving D-finiteness, we have decided to restrict the search to models with finite group.

Certain sufficient conditions have been used in 2D and 3D for proving that the groups for certain models are infinite [11, 14]. However, checking these conditions is expensive, and, as they are just sufficient but not necessary, carrying out these expensive calculations may not be conclusive. We have chosen a more pragmatic approach. Starting from $H=\{\mathrm {id}\}$, we set $H \leftarrow H \cup \bigcup _{i=1}^D \{\Phi _i\circ h:h\in H\}$ until either *H* stabilizes (then *H* is equal to the full group and the group is finite), or the size of *H* exceeds 800 (then we give up and discard the model). The bound 800 was chosen as a compromise between reasonable computing time and reasonable confidence that larger groups are in fact infinite.

## Results

Out of the 7005847194 step sets with cardinality at most 7 or at least 73, there were 58 step sets which survived all the filters specified above, the last filter being, by far, the strongest one. The surviving models are listed at the end of the paper. They all have cardinality 5 or 7.

For models with a finite group, the orbit sum method is one approach to showing that the generating function is D-finite. It rests on the observation that, when certain technical conditions are satisfied, the generating function for a model can be expressed as:

$$\begin{aligned}&f(x_1,\dots ,x_D,t)\\&\quad = \frac{1}{x_1\cdots x_D}[x_1^>\dots x_D^>]\frac{1}{1-t P_S}\sum _{g\in G} {\text {sgn}}(g) g(x_1\cdots x_D), \end{aligned}$$

where *G* is the group, $P_S$ is the step set polynomial as introduced above, $[x_1^>\dots x_D^>]$ is the positive part extraction operator, and ${\text {sgn}}(g)$ refers to the sign of the group element *g*. Note that the expression to which the positive part extraction operator is applied is a rational function. By the closure of D-finiteness under taking positive parts, the formula above implies that the generating function is D-finite.

### Example 2

The generating function *f* of walks in $\mathbb {Z}$ that start at 0, take their steps from $S=\{-1,1\}$, and never leave the non-negative half-line $\mathbb {Z}_{\ge 0}$ satisfies the functional equation:

$$\begin{aligned} (1-tP_S)x f(x,t) = x - tf(0,t). \end{aligned}$$

The step polynomial $P_S(x) = x^{-1} + x$ can be associated with a group *G*. It is finite and given by $G = \{ x\mapsto x,x\mapsto x^{-1} \}$ and acts on the equation above. The second group element transforms the equation above to:

$$\begin{aligned} (1-tP_S)x^{-1}f(x^{-1},t) = x^{-1} - tf(0,t). \end{aligned}$$

Subtracting the two functional equations gives:

$$\begin{aligned} xf(x,t) - x^{-1}f(x^{-1},t) = \frac{1}{1-tP_S} (x-x^{-1}), \end{aligned}$$

and since the series $x^{-1}f(x^{-1},t)$ only involves powers of *x* with negative exponents, it follows that:

$$\begin{aligned} f(x,t) = \frac{1}{x} [x^>] \frac{1}{1-tP_S}(x-x^{-1}). \end{aligned}$$

As the rational function $\frac{x-x^{-1}}{1-tP_S}$ is D-finite and D-finiteness is preserved by taking positive parts, it follows that *f*(*x*, *t*) is D-finite. Incidentally, in this particular example, *f*(*x*, *t*) is even algebraic, because the positive part with respect to a single variable of a rational function can be shown to be always algebraic. In the case of several variables, however, in particular for models in dimension four, the positive part of a rational function is still D-finite but in general not algebraic.

For 50 of the 58 step sets identified by the procedure of Sect. 2, the orbit sum $\sum _{g\in G} {\text {sgn}}(g) g(x_1\cdots x_D)$ happens to be zero. In this case, the “technical conditions” alluded to above are not satisfied and we cannot directly conclude D-finiteness. In the other eight cases, we have checked with Yatchak’s algorithm [21] that the technical conditions are satisfied, so the generating functions of these models are D-finite.

For the 50 cases whose orbit sum is zero, we have tried to detect recurrence equations or differential equations via automated guessing, as systematically done in [8] for 3D models. As remarked in the introduction, we were only able to compute 700 terms for each of these counting sequences, which only in one case (number 13 in the listing below) was enough to find equations. For the generating function of walks with arbitrary endpoint, $f(1,\dots ,1,t)$, we found a linear differential equation of order 12 with polynomial coefficients of degree up to 135. Its coefficient sequence appears to satisfy a linear recurrence of order 18 with polynomial coefficients of degree up to 113.

We suspect that further models are D-finite, but only satisfy equations that are too large to be recovered from 700 sequence terms, and we invite the lattice walk counting community to have a closer look at these models. In the tables below, we write $\bar{1}$ instead of $-1$ for better readability. We also use a pictorial description of the step sets, extending similar descriptions used in the literature for lower dimensions. A step $(s_1,s_2,s_3,s_4) \in \{-1,0,1\}^4$ is represented by a bullet at position $(s_1,s_2,s_3,s_4)$, where $s_1$ is the column block ($-1=\mathrm {left}$, $0=\mathrm {middle}$, $1=\mathrm {right}$), $s_2$ is the row block ($1=\mathrm {top}$, $0=\mathrm {middle}$, $-1=\mathrm {bottom}$), and $s_3,s_4$ are the column and row, respectively, within the block specified by $s_1,s_2$. Models with non-zero orbit sum are highlighted. The orbit sums are stated in a separate table.

## Higher Dimension

Our experiments confirm a trend that was already observed in the investigations of lattice walks in 3D: the number of cases with low dimension, with a Hadamard decomposition, or with a finite group is relatively low. If we are interested in the models which have no Hadamard decomposition, have full dimension, but have a finite group, this means that the Hadamard filter and the dimension filter are relatively weak, while the group size filter is relatively strong. We conclude the paper with three propositions which show that this trend continues (and in fact, quite heavily) when the dimension grows.

### Proposition 3

If *L*(*D*) is the number of step sets $S\subseteq \{-1,0,1\}^D\setminus \{(0,\dots ,0)\}$ whose dimension is less than *D*, then we have $$L(D)\le \left( {\begin{array}{c}3^D-1\\ D-1\end{array}}\right) 2^{\frac{2}{3} 3^D}$$. In particular, $\lim _{D\rightarrow \infty } L(D)/2^{3^D-1}=0$.

### Proof

We first show that for every step set $S\subseteq \{-1,0,1\}^D$ whose dimension is less than *D*, there is a hyperplane $H\subseteq {\mathbb {R}}^D$ generated as linear subspace by elements of $\{-1,0,1\}^D$, such that all elements of *S* point to the same side of *H*. Indeed, consider the convex cone generated by *S* in ${\mathbb {R}}^D$. If $S=\{s_1,\dots ,s_m\}$ does not have full dimension, then there exists an $i\in \{1,\dots ,D\}$, such that for all $(x_1,\dots ,x_m)\in {\mathbb {R}}^m$ with $x_1,\dots ,x_m\ge 0$, the vector $x_1s_1+\cdots +x_ms_m$ cannot have a negative *i*th component unless one of the other components is negative, as well. This implies that the cone generated by *S* is not all of ${\mathbb {R}}^D$. The cone, therefore, has facets, and we can take any hyperplane containing any of its facets as *H*.

To complete the proof, we now bound the number of step sets contained in a hyperplane generated by elements of $\{-1,0,1\}^D$. First, it is clear that there are no more than $$\left( {\begin{array}{c}3^D-1\\ D-1\end{array}}\right) $$ such hyperplanes. Second, each particular hyperplane limits the choice of steps to roughly half of $\{-1,0,1\}^D$, more precisely to at most $\frac{2}{3}3^D-1$ steps. Combining both counts gives the announced bound. $\square$

### Proposition 4

If *H*(*D*) is the number of step sets $S\subseteq \{-1,0,1\}^D\setminus \{(0,\dots ,0)\}$ which admit a Hadamard decomposition, then we have $H(D)\le (D-1)2^{\frac{2}{3}(3^{D}+1)}$. In particular, $\lim _{D\rightarrow \infty } H(D)/2^{3^D-1}=0$.

### Proof

For a $d\in \{1,\dots ,D-1\}$, a step set *S* is *d*-Hadamard if there are $V,U\subseteq \{-1,0,1\}^d$ and $W\subseteq \{-1,0,1\}^{D-d}$, such that $S=(V\times \{0\})\cup (U\times W)$. There are $2^{3^d}$ choices for *V* and *U* and $2^{3^{D-d}}$ choices for *W*, which makes $2^{2\,3^d+3^{D-d}}$ combinations (*V*, *U*, *W*) for a specific *d*. The total number of Hadamard models is, therefore, bounded by $\sum _{d=1}^{D-1} 2^{2\,3^d+3^{D-d}} \le (D-1) 2^{\frac{2}{3}(3^D+1)}$. $\square$

Estimating the number of models with finite group is slightly less elementary. The idea is to reduce the problem to the case of weighted models in the quarter plane. In a weighted model, each element of the step set has an element of an integral domain *A* attached to it. In the step set polynomial, these elements appear as coefficients of the terms. For example, $5x_1^{-1}x_2 - 3x_1 + 7x_2 + x_1x_2^{-1}$ is the step set polynomial of the model in which the step $(-1,1)$ has weight 5, the step (1, 0) has weight $-3$, etc. The group of a weighted model is defined in the same way as for unweighted models. In [18], it was asked which choices of weights lead to which groups, and it was found that weight vectors leading to a specific group form an algebraic variety. From general results about groups on elliptic curves [16, Remark 5.1], it follows that only finitely many different groups can arise as groups of a weighted walk (namely, the dihedral groups with 4, 6, 8, 10, 12, or infinitely many elements). As there is an algebraic variety associated with each of the finite groups, and the union of finitely many algebraic varieties is again an algebraic variety, we can conclude that there exists a non-zero polynomial:

$$\begin{aligned} Q\in {\mathbb {Z}}[z_{-1,-1},z_{-1,0},z_{-1,1},z_{0,-1},z_{0,0},z_{0,1},z_{1,-1},z_{1,0},z_{1,1}], \end{aligned}$$

such that for all weight vectors $(a_{-1,-1},\dots ,a_{1,1})$ that correspond to a weighted model with a finite group, we have $Q(a_{-1,-1},\dots ,a_{1,1})=0$. We will use this observation in combination with the following lemma to show that models with finite groups are rare.

### Lemma 5

(“Zippel’s Lemma”; Proposition 97 in [22]) Let *A* be an integral domain, $Q\in A[z_1,\dots ,z_n]$ and the degree of *Q* in each variable $z_i$ be bounded by $\delta$. Let *Z*(*k*) be the number of zeroes $(\zeta _1,\dots ,\zeta _n)$ of *Q* where each $\zeta _i$ is restricted to a set with *k* elements, for some $k\gg \delta$. Then, $Z(k)\le n\delta k^{n-1}$.

### Proposition 6

If *F*(*D*) is the number of step sets $S\subseteq \{-1,0,1\}^D\setminus \{(0,\dots ,0)\}$ whose associated group is finite, then we have $F(D)\le c 2^{\frac{8}{9} 3^D}$, for some constant *c*. In particular, $\lim _{D\rightarrow \infty } F(D)/2^{3^D-1}=0$.

### Proof

Consider a step set $S\subseteq \{-1,0,1\}^D$ with a finite group. Write its step set polynomial as:

$$\begin{aligned} P_S=\sum _{i,j=-1}^1 p_{i,j}(x_3,\dots ,x_D)x_1^ix_2^j. \end{aligned}$$

Since the group of *S*, which is generated by the involutions $\Phi _1,\dots ,\Phi _D$, is finite, so is in particular the subgroup generated by the involutions $\Phi _1$ and $\Phi _2$. This subgroup is equal to the group of the weighted 2D model in which the weight associated with a step $(i,j)\in \{-1,0,1\}^2$ is the polynomial $p_{i,j}(x_3,\dots ,x_D)$. Therefore, if *Q* is the polynomial discussed above, we have $Q(p_{-1,-1},\dots ,p_{1,1})=0$.

In summary, we have shown so far that there are no more step sets in $\{-1,0,1\}^D$ with finite group than there are weighted models with finite group and with step set in $\{-1,0,1\}^2$ whose weights are step set polynomials in $D-2$ variables. There are $k=2^{3^{D-2}}$ such polynomials. By Lemma 5, there is a constant $\delta$ (bounding the degree of the polynomial *Q*), such that *Q* has at most $9\delta (2^{3^{D-2}})^{9-1}=9\delta 2^{\frac{8}{9} 3^D}$ roots whose coordinates all are such polynomials. Therefore, there are at most so many models in *D* dimensions with a finite group. $\square$

## Tables

See Tables 1, 2, 3, and 4

**Table 1:**
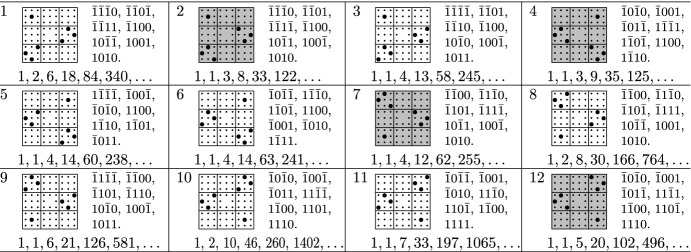
Models with a group isomorphic to $C_2\times C_2\times S_3$

**Table 2:**
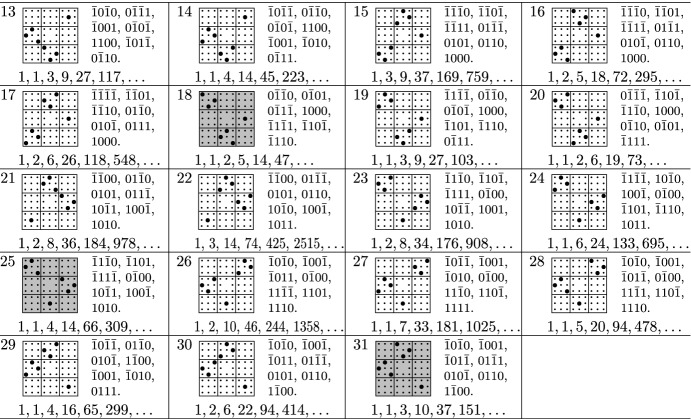
Models with a group isomorphic to $S_3\times S_3$

**Table 3:**
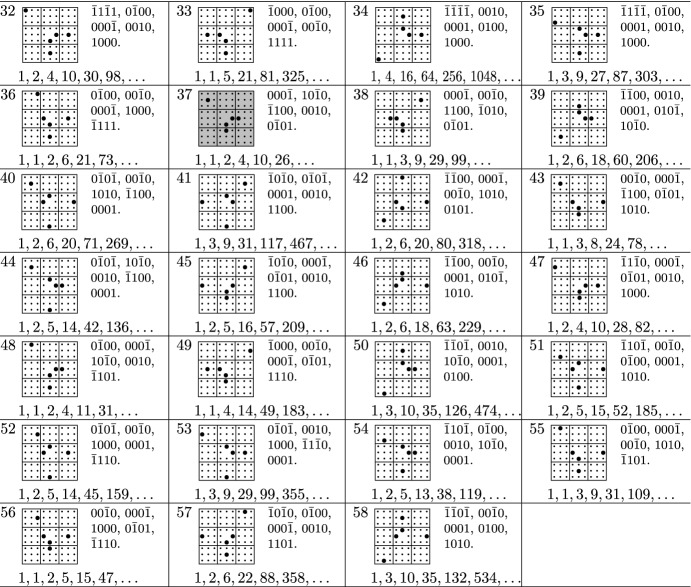
Models with a group isomorphic to $S_5$

**Table 4:** Non-zero orbit sums

idx	Orbit sum
4	${\frac{(w^2 - z) (w z-1 ) (w - z^2) (w^2 + z - w y^2 z + w z^2) (w^2 x^2 - w y + x^2 z - w^2 y z + w x^2 y^2 z + w x^2 z^2 - y z^2)}{w^3 x y z^3 (w^2 + z + w y^2 z + w z^2)}}$
2	${\frac{(w^2 - z) ( w z-1 ) (w - z^2) (w + w^2 z - w y^2 z + z^2) ( w^2 x^2 y - w^2 z + x^2 y z - w y^2 z - z^2 + w x^2 y z^2 - w )}{w^3 x y^2 z^3 (w^2 + z + w z^2)}}$
7	${\frac{(w^2 - z) (1 - w z) (w - z^2) (w^2 x^2 y - w y^2 - w z + x^2 y z - w^2 y^2 z + w x^2 y z^2 - y^2 z^2) (w y^2 - w z + w^2 y^2 z + y^2 z^2)}{w^2 x y^2 z^2 (w + w^2 z + z^2) (w^2 + z + w z^2)}}$
12	${\frac{(w^2 - z) (1 - w z) (w - z^2) (w^2 y^2 - w z + y^2 z + w y^2 z^2) (w^2 x^2 y^2 + w x^2 z - w^2 y z + x^2 y^2 z - y z^2 + w x^2 y^2 z^2-w y )}{w^2 x y z^2 (w^2 + z + w z^2) (w^2 y^2 + w z + y^2 z + w y^2 z^2)}}$
18	${\frac{(w^2 - z) (1 - w z) (w - z^2) (w + w^2 z - w x y z + z^2) (w^2 x + x z - w y^2 z + w x z^2) (w^2 x^2 - w y + x^2 z - w^2 y z + w x^2 z^2 - y z^2)}{w^4 x^2 y^2 z^4 (w^2 + z + w z^2)}}$
25	${\frac{(w^2\! - z) (1\! - w z) (w\! - z^2) (w^2 x^2\! - w y + x^2 z - w^2 y z + w x^2 z^2\! - y z^2) (w^2 x y - w z + x y z + w x y z^2) (w y^2 - w x z + w^2 y^2 z + y^2 z^2)}{w^2 x^2 y^2 z^2 (w + w^2 z + z^2) (w^2 + z + w z^2)^2}}$
31	${\frac{(w^2 - z) ( w z-1 ) (w - z^2) (w y - w x^2 z + w^2 y z + y z^2) ( w^2 x y - w^2 z + x y z - z^2 + w x y z^2-w ) (w^2 y^2 - w x z + y^2 z + w y^2 z^2)}{w^3 x^2 y^2 z^3 (w^2 + z + w z^2)^2}}$
37	${\frac{ (w^2 - y) ( w y-x ) (w x - y^2) (w x - z) ( w z-1) ( w z-x y) ( x z-y) (w - y z) (x^2 - y z) (x - z^2)}{w^4 x^4 y^4 z^4}}$
